# In vitro tear replenishment system: assessing drug delivery from contact lens biomaterials through corneal epithelial monolayer and multilayer under replenishment conditions

**DOI:** 10.1007/s13346-024-01746-z

**Published:** 2024-12-05

**Authors:** Saman Mohammadi, Shahabedin Eslami, Lyndon Jones, Maud Gorbet

**Affiliations:** 1 Systems Design Engineering, University of Waterloo, 200 University Ave W, Waterloo, ON N2L 3G1 Canada; 2 Centre for Ocular Research and Education (CORE), School of Optometry and Vision Science, University of Waterloo, 200 University Ave W, Waterloo, ON N2L 3G1 Canada

**Keywords:** Ocular drug delivery, Contact lens, In vitro ocular model, Corneal epithelial cells, Tear replenishment

## Abstract

There is a need to develop improved in vitro ocular models for biocompatibility and drug delivery studies to assess the potential of in vivo performance of contact lenses. By using an in vitro corneal epithelial cell model combined with a tear replenishment method, this study aimed to investigate the delivery of the glaucoma drug latanoprost from contact lenses and compare the dynamic release results to no-replenishment (immersion) conditions. Corneal epithelial cells were grown as a monolayer or multilayer on curved cellulose cell culture inserts. Three contact lens materials (balafilcon A; senofilcon A; etafilcon A), soaked for 24 h in latanoprost, were placed on the curved cornea models (CCM) and drug concentration was determined on the basal (diffusion/transport) and apical (supernatant) sides after 1, 4, 8 and 12 h. The in vitro tear replenishment was achieved via intermittent flow of a tear solution over the CCM at a rate of 1 mL/hour. A zero-order kinetic was observed for basal drug concentration over the 12 h period. Similar basal and apical drug concentrations were observed with monolayer and multilayer CCM, except for the etafilcon A material. The apical release of latanoprost was significantly lower under replenishment compared to no-replenishment conditions. These results demonstrate the role that a dynamic release model will have in predicting the amount of drug that can be released from a contact lens into the tear film and the critical role of a cell monolayer in in vitro drug delivery studies.

**Graphical Abstract:**

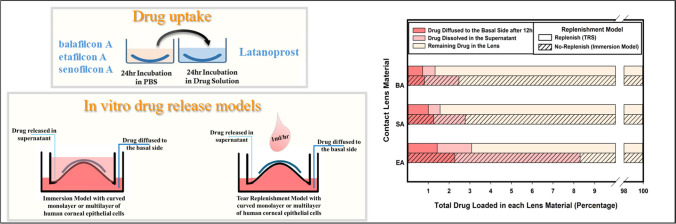

## Introduction

With the increasing interest in using contact lens as drug delivery devices as well as the desire to understand the complex interactions between contact lens disinfecting and cleaning solutions and lenses, there has been a need for the development of enhanced in vitro cornea models that can assess contact lens drug delivery systems and biocompatibility under dynamic conditions to help better identify promising candidates for future clinical studies [1–3]. In vitro human cornea models offer a cost effective and more standardizable substitute to animal studies, which can be costly and may be difficult to interpret given both interspecies variation and animal-to-animal inconsistency [4–9]. Several cell culture models of the cornea and corneal epithelium have been used to study in vitro ocular toxicity and permeability [10–13]. Reconstructed cornea equivalents, which include all corneal layers have been shown to correlate reasonably well with in vivo results [14]. However, it is recognized that the epithelium, through its tight junctions, acts as the primary barrier against transcorneal permeation and is also responsible for 99% of the corneal electrical resistance [15–18]. The corneal epithelium alone could thus be considered as a good proxy for an in vitro ocular drug model for contact lens delivery as it can reduce cell culture cost and time, and allow for higher throughput testing of biomaterial interactions and drug permeation [12]. Furthermore, besides reproducing the barrier function of the epithelium in vitro, corneal epithelial cells express several membrane receptors and also contain enzymes, both of which are involved in transport and metabolization of prodrugs, such as latanoprost, a widely-used anti-glaucoma medication [19–23]. Human corneal epithelial cells express high levels of prostaglandin transporters OATP2A1 (Organic Anion Transporting Polypeptide 2A1) which contributes to the transcellular transport of latanoprost, a prostaglandin F2*α* analogue [19].

To evaluate ocular drug delivery systems and new drugs, in vitro cell models such as Franz and Bronaugh cells have been used [13, 14, 24–27]. In a Franz cell, the drug solution diffuses from the donor chamber through the excised corneal tissue or an in vitro cornea model into the receptor chamber where samples of the drug are collected. The stirring prevents formation of a stagnant boundary transfer layer. While steady-state permeability experiments enable assessment of corneal permeability, metabolism and active transport of the drug, it does not account for the rapid elimination of the drug in the tear film. Understanding the pre-corneal elimination kinetics, as well as distribution and elimination in the anterior chamber of the eye, when combined with permeability studies may lead to the development of optimized drug delivery systems and the successful simulation of in vivo drug diffusion through the cornea [28]. Similar to the in vitro ocular permeability models, in vitro biocompatibility and toxicology models currently do not remove/replenish nutrients on the corneal epithelial cells during incubation, and thus cells are exposed to the same concentration over an extended period of time [29–33].

An in vitro tear replenishment system (TRS) was previously developed to deliver a tear solution analogue to an in vitro ocular cell culture model and the ophthalmic material interacting with the ocular cell model while maintaining the air–liquid interface [34]. However, it was observed that maintaining higher flow rates with the “atomized jet design” could damage the integrity of the cornea model over long periods of time and thus prevented its use for biocompatibility and drug delivery studies [34]. The TRS was redesigned to include (1) a dripping flow regime which allowed for longer time study and (2) the collection of the supernatant from each well/ocular model for analysis. The new TRS model, presented here, was used to investigate the effect of tear replenishment on the release of latanoprost from three commercially available contact lens materials. Latanoprost was chosen as the model drug in this study, as the hydrolysis of latanoprost prodrug by corneal epithelial cells before diffusion through the cornea is an important factor to consider in evaluating drug delivery systems in vitro [19, 21, 35, 36]. In this study, drug delivery from contact lens materials was assessed for 12 h in a corneal epithelial cell model (monolayer and multilayer) with and without tear replenishment.

## Materials and Methods

### In Vitro Tear Replenishment Model

**Fig. 1:**
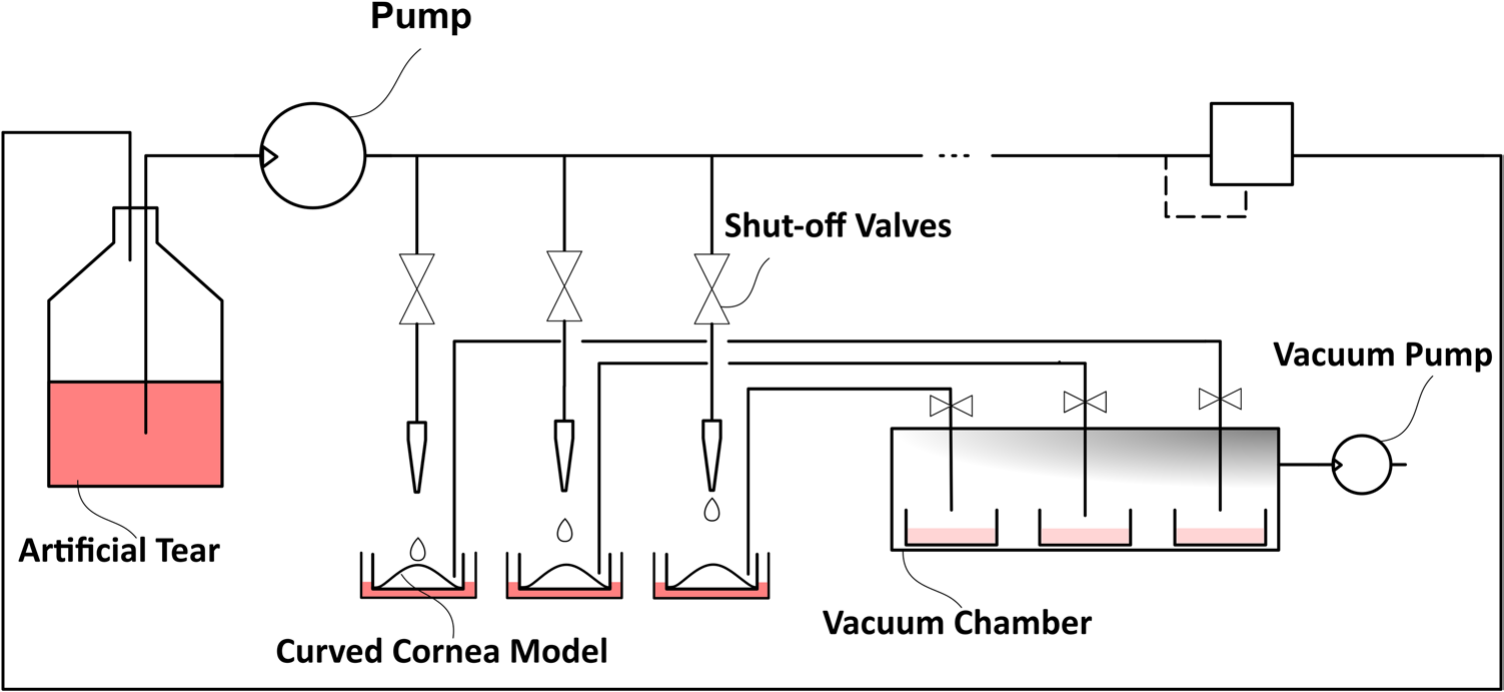
Schematic of the tear replenishment system. A solenoid pump provides enough back pressure to a series of isolation valves to control the flow rate as well as the duty cycle of the tear drop over the curved cornea model. An air pump creates a relative vacuum which drains the supernatant from the top of each corneal cell model and collects them individually. Up to six curved corneal cell models can be tested in parallel

To generate a dripping flow, a stainless-steel micro-needle with an internal diameter of 100* µm* was used (Hamilton Company^®^, Nevada, USA) to reliably deliver tear solution analogue without the risk of clogging the needle while maintaining a flow rate as low as possible. Using the dripping flow and a frequency of 10 drops per minute, the total flow rate of the TRS was 1 *mL*/hour, which, while relatively small, was albeit 10 times greater than the physiological tear replenishment rate. The microfluidic system, as shown in Fig. 1, used a pressurized supply line and the tear solution analogue was transferred through a needle above the surface of the in vitro curved cornea model before dripping onto the surface of the cornea model, which could be covered with a contact lens. The amount of delivered solution was controlled through a series of solenoid isolation valves (Bio-Chem Fluidics, NJ, USA) similar to the former design [34]. The air–liquid interface of the cornea model was maintained by automatically collecting/draining the supernatant through another solenoid-operated pinch valve into the well of a 6-well plate encased in the vacuum chamber as part of the supernatant drainage module.

**Fig. 2:**
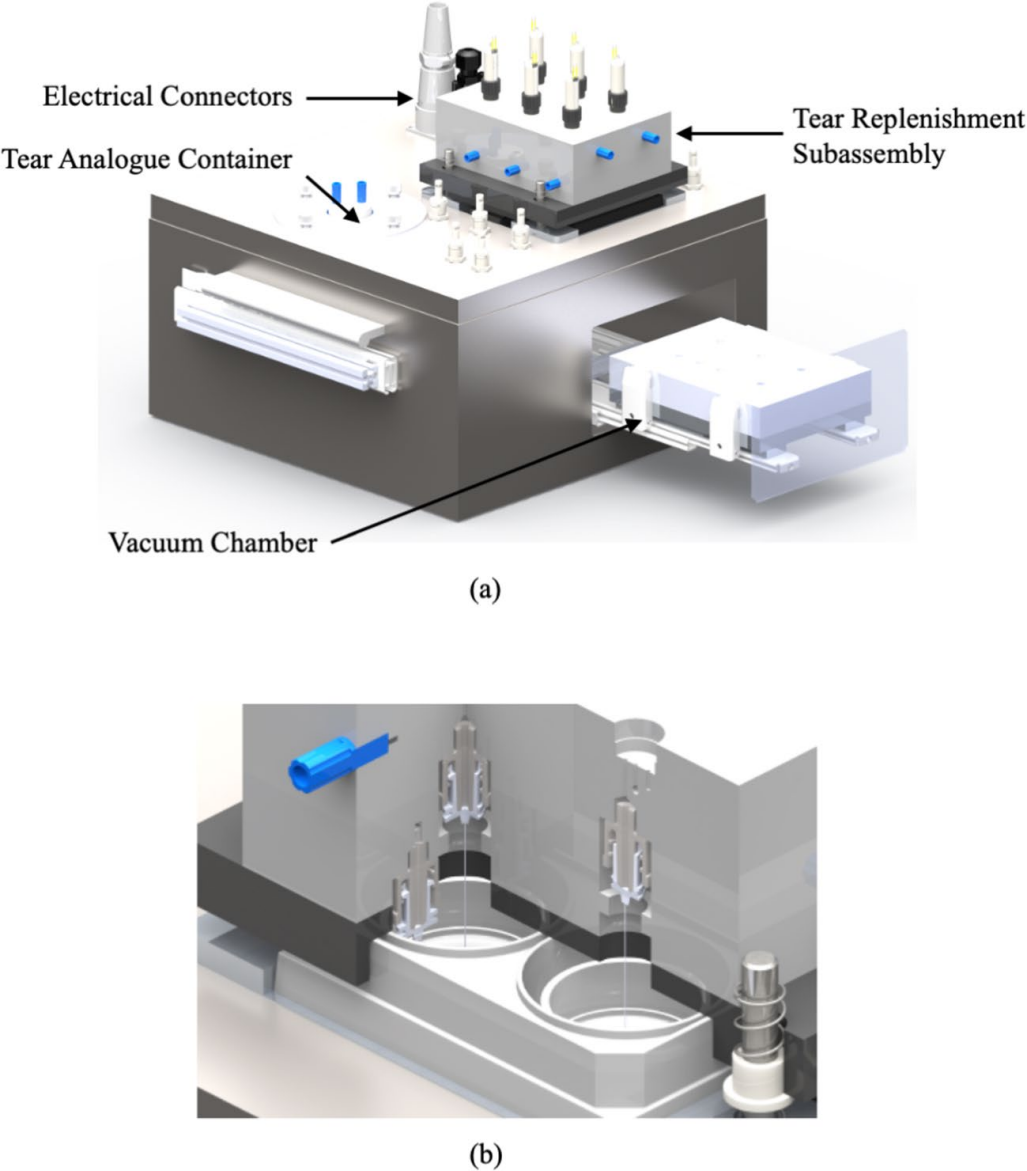
**a**) Assembled 3D-model of the tear replenishment system (TRS) and its various components. **b**) Schematic cross section of the TRS with the nozzle assembly and the stratified curved cornea model

The mechanical components of the TRS were divided into the tear replenishment module and the supernatant drainage module. While the tear replenishment module was required to be accessible to place/remove the cell culture plate containing the curved cornea models, the supernatant module, along with the rest of electro-mechanical components were encased in a stainless-steel enclosure. A solenoid operated micro-pump from Bio-Chem Fluidics™ was used to pump the tear solution analogue through PTFE tubing (Bio-Chem Fluidics, NJ, USA). Figure 2 shows the designed system and the two modules.

**Fig. 3:**
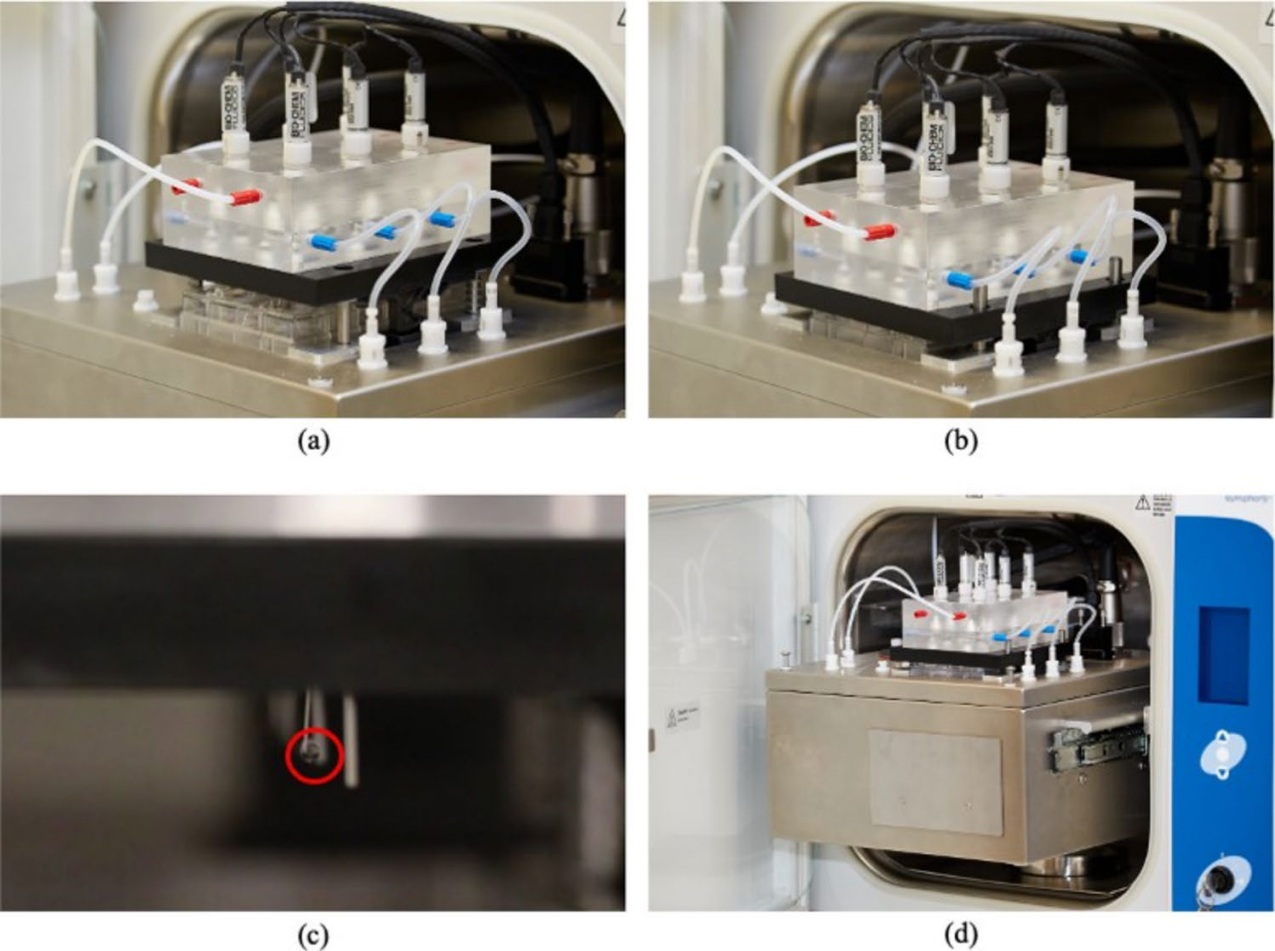
Tear Replenishment System Design. A 6-well plate with 6 corneal cell models is placed inside the replenishment subassembly **a**) before, **b**) after clamping the subassembly to seal the 6-well plate; **c**) Tear analogue dripping from micro-needle **d**) The TRS is designed to fit in a cell culture incubator so that cells are exposed to the proper growing environment

An air jacketed CO_2_ mini-incubator (VWR International, Mississauga, Canada) was adapted to enclose the tear replenishment system and provide an ideal cell culture environment (100% humidity, 37^◦^*C* temperature, and 5% CO_2_). All the electrical components were located in the electrical panel of the incubator. The electro-mechanical components were passed through the incubator compartment using a waterproof, chemically resistant connector. Relocating electrical components also eliminated an extra heat source that would destabilize the incubation temperature. All the components used in the device were sterilizable and biologically inert. The implemented tear replenishment system as well as the interior of the device are presented in Fig. 3.

### Latanoprost Doping Solution

The lens doping solutions were prepared by dissolving latanoprost (solution in methyl acetate, Cayman Chemical, Ann Arbor, MI, USA) in PBS (Lonza, Walkersville, MD, USA). The concentration of the high dose stock drug solution was measured at 123* µg/mL*. A low dose stock solution of 13* µg/mL* was also prepared.

### Preparation of Contact Lenses

Based on results obtained previously [35], three commercially available contact lens materials, senofilcon A, etafilcon A, and balafilcon A were selected as potentially suitable (and differing) drug carrying hydrogels. The properties of the three lens types are presented in Table 1. All lenses had a back vertex power of −3.00 diopter. Lenses were incubated for 24 h in PBS (Lonza, Allendale, NJ, USA) to remove any remnants of their packaging solutions, prior to incubation in 1*.*0* mL* of the drug solution for 24 h.

**Table 1:** Properties of the Contact Lens Hydrogel Materials Used in the Tear Replenishment System

Commercial name (US adopted name)	Acuvue 2 etafilcon A	Acuvue Oasys^†^ senofilcon A	PureVision 2 ^‡^ balafilcon A
Manufacturer	Johnson & Johnson	Johnson & Johnson	Bausch & Lomb
Water content (WC)	58	38	36
Principal monomer	HEMA + MA	mPDMS + DMA + HEMA + siloxane macromer + TEGDMA + PVP	NVP + TPVC + NVA + PBVC
FDA group	IV High WC Ionic	V(I) Low WC Non-ionic	V(III) Low WC Ionic

^†^ An internal wetting agent (PVP) is incorporated to overcome the hydrophobicity at the surface of this silicone hydrogel material. ^‡^ Plasma oxidation process has been used as a surface treatment to increase wettability

*HEMA*, HydroxyEthyl MethaAcrylate; *MA*, Methacrylic Acid; *mPDMS*, monofunctional PolyDiMethylSiloxane; *DMA*, DiMethAcrylate; *TEGDMA*, Tetra-EthyleneGlycol DiMethAcrylate; *PVP*, Polyvinylpyrrolidone; *NVP*, N- Vinylpyrrolidone; *TPVC*, Tris(trimethylsiloxysilyl) Propyvinyl Carbamate; *NVA*, N-Vinyl Aminobutyric Acid; *PBVC*, Poly(dimethysiloxy)di (silylbutanol) Bis(Vinyl Carbamate)

### In Vitro Corneal Epithelial Cell Models

HPV-immortalized human corneal epithelial cells (HCEC) were cultured in keratinocyte serum-free medium supplemented with bovine pituitary extract, recombinant epidermal growth factor, and Penicillin/Streptomycin (1% Pen/Strep) (KSFM) (ScienCell, Carlsbad, CA, USA) at 37 °C and 5% CO_2_. Fresh medium was added every other day and cells were grown to 90% confluency in tissue culture treated flasks. Adherent cells were removed using TryplExpress dissociation solution (Life Technologies, Burlington, ON, Canada) and resuspended in KSFM prior to seeding.

The curved cornea models (monolayer and multilayer) were grown on a Millicell- HA (mixed cellulose esters) membrane (Millipore, Billerica, MA, USA) with a 0*.*45* µm* pore size as previously described in Postnikoff et al. [10]. In brief, the 30 mm diameter inserts were curved using a custom-shaped mold. Silicone rings (inner diameter of 15.9 mm and an outer diameter of 23.0 mm) were punched from Press-to-Seal sheets with adhesive (Life Technologies, Burlington, ON, Canada) and disinfected with 70% ethanol and placed on top of the curved inserts. The assembled inserts were then UV sterilized. After sterilization, inserts were coated with collagen type I (0*.*05* mg/mL* - 30* min* at 37^◦^*C*). The inserts were then rinsed with PBS before adding cells.

The curved inserts were seeded with 6 × 10^5^ cells/insert. Cells were fed with KSFM on each of the basal and apical sides of the curve for seven days, with medium being exchanged every other day. A confluent monolayer with tight junctions grew under these conditions and was ready for experimentation at day 7.

For multilayer curved cornea models, cell differentiation was induced at day 5 by exposing the monolayer to an air–liquid interface. Cells were fed only on the basal side with 2% fetal bovine serum (FBS, Invitrogen) in 1:1 Dulbecco’s minimum essential medium (DMEM, Invitrogen) in Ham’s F12 nutrient medium (DMEM/F12, Invitrogen); the medium was exchanged daily [10]. The cells grew under these conditions for seven days. The multilayer (stratified) corneal epithelia were then ready for experimentation.

### Drug Release Experiments With and Without Tear Replenishment

The microfluidics system was sterilized by first running 70% ethanol through the device, followed by PBS. The device was then required to stabilize near 37^◦^*C* to eliminate temperature fluctuations as well as condensation on the device components during the experiment. Afterwards, curved cornea models with latanoprost-loaded contact lenses were placed in the device under aseptic conditions, then the entire device was transferred back to the cell culture incubator and experiments were performed for 12 h. KSFM was used as the tear solution. In the no-replenishment conditions, also referred to as “No-Replenish” in the figures, curved cornea models were immersed in 2.5 ml of KSFM. All experiments included controls, curved cornea models without a lens and no-replenishment conditions.

### Measuring Drug Concentrations

Using a sterile pipette, aliquots of 200*µL* (10% of the total volume of the medium present in the bottom) were taken from the bottom of the in vitro curved cornea models (ie on the basal side) and replaced by fresh culture medium for both replenishment and no-replenishment conditions. Samples from the basal side were taken at 1, 4, 8, and 12 h and contained the drug that had been released from the contact lens material on top of the cells, then diffused through the cells (either through active or passive transport) and the insert’s membrane. An aliquot of the supernatant solution (ie apical side) was also taken at 12 h from the insert for the “No-Replenish” samples and from the collection well in the drainage module for the “Replenish” samples. Collected samples were analyzed by an enzyme immuno-assay (EIA) for latanoprost (Cayman Chemical, Ann Arbor, MI, USA). According to the EIA kit instructions, each collected sample was analyzed at four different dilutions. To determine the uptake amount by the contact lenses, samples were also aliquoted from the original drug stock solution as well as the remaining drug solutions after soaking the lenses. It should be noted that the EIA kit does not distinguish between the free-acid form and ester form of the drug.

### Cell Viability Assay

After 12 h of drug release in the curved cornea models, contact lenses and medium were removed and cellular viability was assessed using the MTT assay as previously described [10]. Dimethyl thiazoyl blue tetrazolium bromide (0*.*5* mg/mL*, MTT, Sigma Aldrich, Oakville, ON, Canada) was added to the apical and basal sides of the cell culture insert and incubated for 3 h at 37^◦^*C* and 5% CO_2_. The MTT solution was then removed and isopropanol was added to both the apical and basal sides of the insert and plates were agitated for 2 h. The solutions in the apical and basal sides were mixed together and samples were read in a UV–Vis spectrophotometer at an optical density of 595 nm with a reference at 650 nm. All results are expressed as the relative viability compared to control cells: cells incubated in the absence of a contact lens and with no-replenishment, an immersion model.

### Real-Time PCR

Total RNA from HPV-immortalized human corneal epithelial cells grown to confluence in a 25*cm*^2^ flask was extracted with an RNA isolation kit (Illustra RNAspin Mini; GE Healthcare, Little Chalfont, Buckinghamshire, UK) according to the kit’s protocol. RNA samples were stored at *-*80^◦^*C* until analysis. Reverse transcription of extracted RNA was performed using a Superscript III kit (Invitrogen, Carlsbad, CA, USA). The cDNA was amplified in a sequence detection system (ABI 7900HT; Applied Biosys- tems, Bedford, MA, USA) under the following conditions: 45 cycles of 50^◦^*C* for 2 min, followed by denaturation at 95^◦^*C* for 15 s, and then annealing at 60^◦^*C* for 1 min. Predesigned primers and TaqMan probes for SLCO2A1 (OATP2A1) and the housekeeping genes *β*-actin (ACTB) and glyceraldehyde 3-phosphate dehydrogenase (GAPDH) were used (Applied Biosystems) [19, 37]. Amplified PCR cDNA samples were mixed with bromophenol blue and then loaded on the agarose gel. Electrophoresis was run at 85 V and 300 mA until the samples had migrated to within 3*/*4 of the end of the gel. The gel was then removed and photographed under a UV lamp.

### Inhibition of Transporters and Metabolizing Enzymes

To gain a further understanding of the role of cells in the transport of the drug released by the contact lens in our in vitro model, Diclofenac was used to inhibit the prostaglandin transporter OATP2A1/SLCO2A1 [37]. Diclofenac sodium salt (Sigma- Aldrich) was dissolved in KSFM at 100 µM and corneal epithelial monolayers were incubated with the diclofenac solution for 24 h prior to the experiments [37]. Additionally, to assess the contribution of active transport and metabolism, corneal epithelial monolayers were fixed using 2% paraformaldehyde for 24 h prior to the experiments. Experiments with latanoprost-loaded balafilcon A and senofilcon A were performed under no-replenishment conditions and samples were collected from the basal side at 12 h and processed as described above.

### Statistical Analysis

For all studies, a minimum of three experiments were performed on different dates. Results are reported as the mean of at least three experiments ± standard deviation. For both cell viability and drug release results, to evaluate the significance of the differences, an analysis of variance (ANOVA) was performed, followed by multiple pair-wise comparisons using the Holm-Sidak test and a pairwise comparison using T tests according to Sidak correction of Bonferroni inequality in SigmaPlot™.

## Results

### Cell Viability

**Fig. 4:**
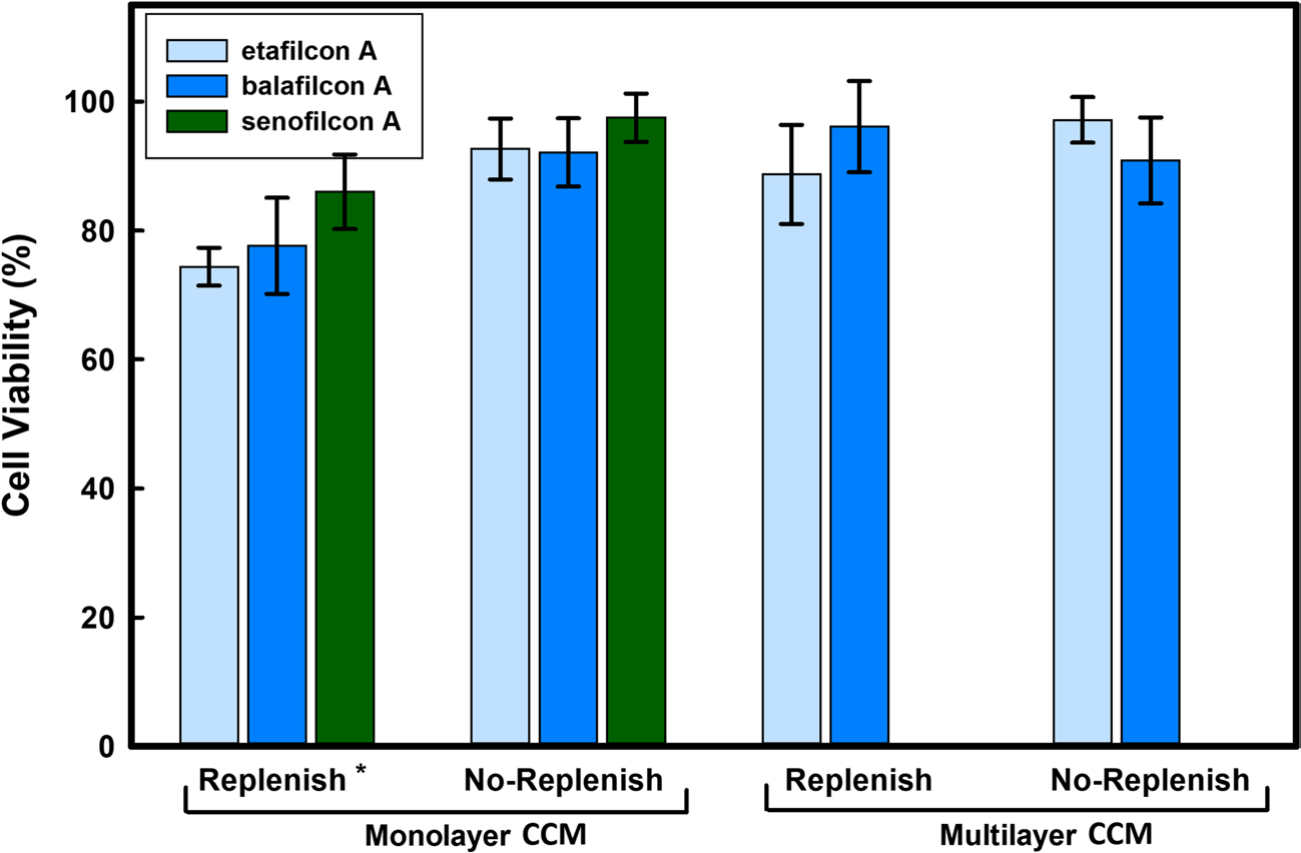
Corneal epithelial cell viability after the 12-h release study with and without replenishment in monolayer and multilayer curved cornea models. No-replenish indicates that models were immersed in medium for 12 h while Replenish indicates that the models were exposed in the TRS to a flow rate of 1 mL/h with automatic drainage to prevent immersion. Viability was measured by an MTT assay and is expressed as a percentage relative to in vitro curved cornea model (CCM) with no-replenishment and without a contact lens. The results represent the mean of three experiments (n = 3 ± standard deviation). TRS: Tear Replenishment System. ^∗^ Significantly different from no-replenish condition and multilayer curved cornea model (*p* = 0*.*002)

A significantly damaged cornea model would invalidate the obtained release results. Cell viability was thus assessed as a means to verify the integrity of the monolayer and multilayer curved cornea models after the drug release experiments; if cells were washed off during replenishment, a lower “viability” would be observed due to loss of cells compared to the control. Due to its purple color, the MTT assay also allowed for a visual inspection of the integrity of cell coverage on the curved inserts. No visible damage to the cornea models was observed after 12 h of drug release experiments. As shown in Fig. 4, the viability remained above 80% in the experiments using the monolayer curved cornea model and above 90% in the multilayer model. In the monolayer curved cornea model, the reduction in viability observed under the replenishment conditions was statistically significant when compared to no-replenishment (*p* = 0*.*002); this was likely due to some cells being sloughed off under the dynamic flow conditions. No significant difference in viability was observed between the lens materials (*p* > 0*.*4).

### Drug Uptake and Release Studies

The high dose latanoprost doping solution’s concentration was measured at 123*.*4 ± 12*.*7* µg/mL* and 13*.*4 ± 1*.*5* µg/mL* in low dose latanoprost solution. The total drug uptake was calculated by measuring the remaining drug in the doping solution before and after incubation of contact lenses and is reported in Table 2.

**Table 2:** High Dose (HD) and Low Dose (LD) Latanoprost Uptake into Three Commercial Contact Lens Materials

Contact lens material	Drug remaining in doping solution (*µg*)	Drug uptake [*µg/lens*]	Uptake as a percentage of available drug (%)
HD-senofilcon A	0*.*163 ± 0*.*048	123*.*24	99*.*9
HD-balafilcon A	0*.*178 ± 0*.*048	123*.*23	99*.*9
HD-etafilcon A	3*.*713 ± 0*.*846	119*.*7	97*.*0
LD-balafilcon A	0*.*122 ± 0*.*057	13*.*31	99*.*0
LD-etafilcon A	0*.*064 ± 0*.*003	13*.*37	99*.*5

Lenses were soaked for 24 h in 1* mL* of drug doping solution (123*.*4* µg/mL* for high dose and 13*.*44* µg/mL* in low dose). Latanoprost concentrations were measured using EIA. (n = 4, Mean ± Standard Deviation)

### Apical and Basal Release of High Dose Latanoprost-Eluting Contact Lenses in a Monolayer Curved Cornea Model

**Fig. 5:**
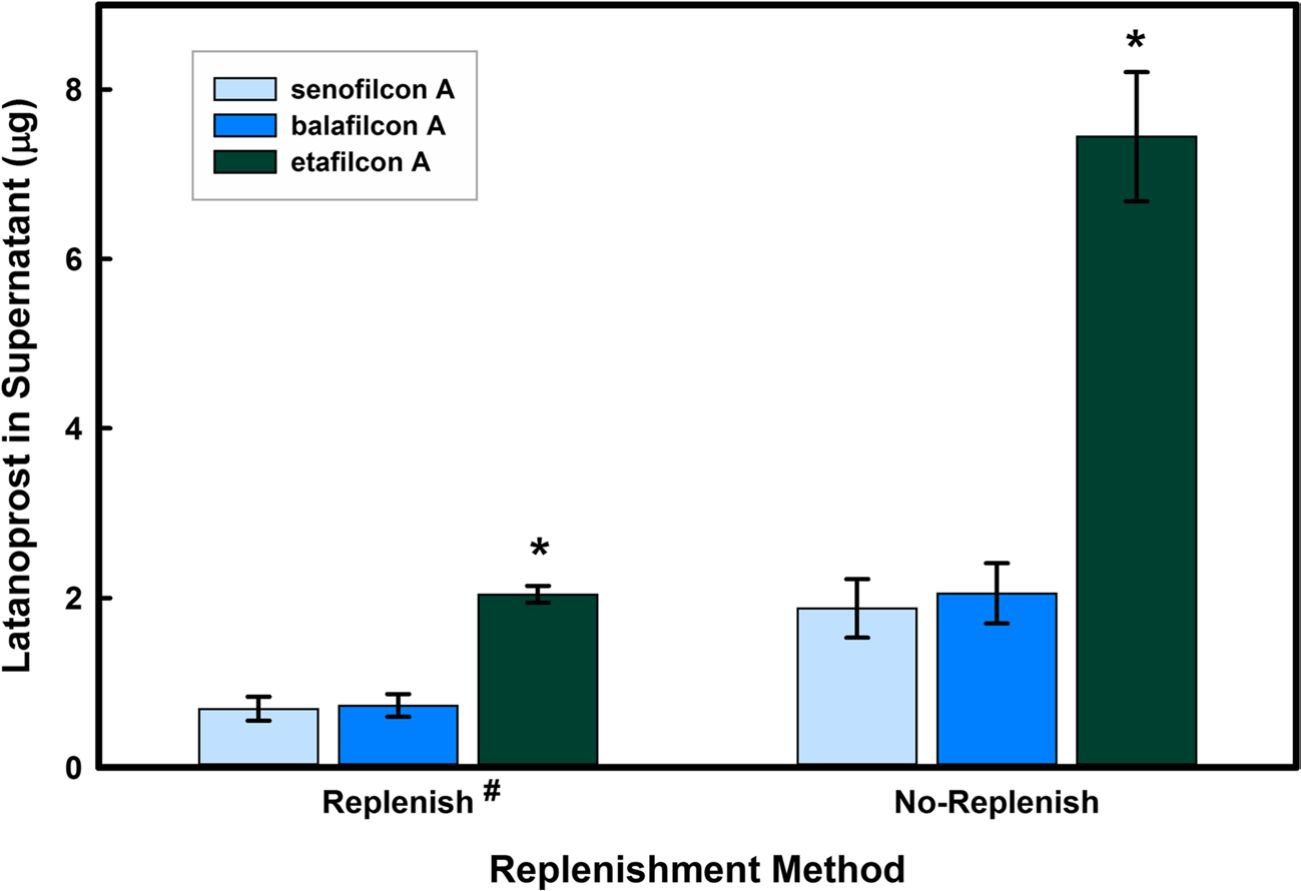
The effect of replenishment on the apical levels of latanoprost following latanoprost release (high dose) from three contact lens materials in a monolayer curved cornea model. Lenses were soaked for 24 h in drug solution (123*.*4* µg/mL*) and then placed on the curved monolayer for 12 h. Aliquots were taken at the end of the experiment from the apical side of the models without replenishment and from the supernatant container for the curved cornea models exposed to replenishment. Drug concentrations were measured using EIA. No-replenish indicates that models were immersed in medium for 12 h while Replenish indicates that the models were exposed in the TRS to a flow rate of 1 mL/h with automatic drainage to prevent immersion. ^#^ Significantly different from no-replenish samples (*p* < 0*.*001). ^∗^Significantly different from silicone hydrogels (*p* < 0*.*005). (n = 3, mean ± standard deviation)

The apical release of the drug under the immersion condition (No-Replenish) is defined as the amount of drug released from the contact lens into the supernatant tear analogue solution on the apical side of the curved cornea model (2*.*5* mL*). Under the tear replenishment (Replenish) conditions, the apical release is defined as the amount of the drug released from the contact lens and collected in the supernatant drainage module (12* mL* total volume). The apical release results, as depicted in Fig. 5, showed that significantly more latanoprost was dissolved in the supernatant under the immersion condition compared to the tear replenishment condition (*p* < 0*.*001), despite a significantly smaller supernatant volume (2*.*5* mL*). Furthermore, the apical release of latanoprost from the conventional lens material etafilcon A was significantly higher compared to the silicone hydrogels (*p* < 0*.*005), while there was no difference between silicone hydrogels.

**Fig. 6:**
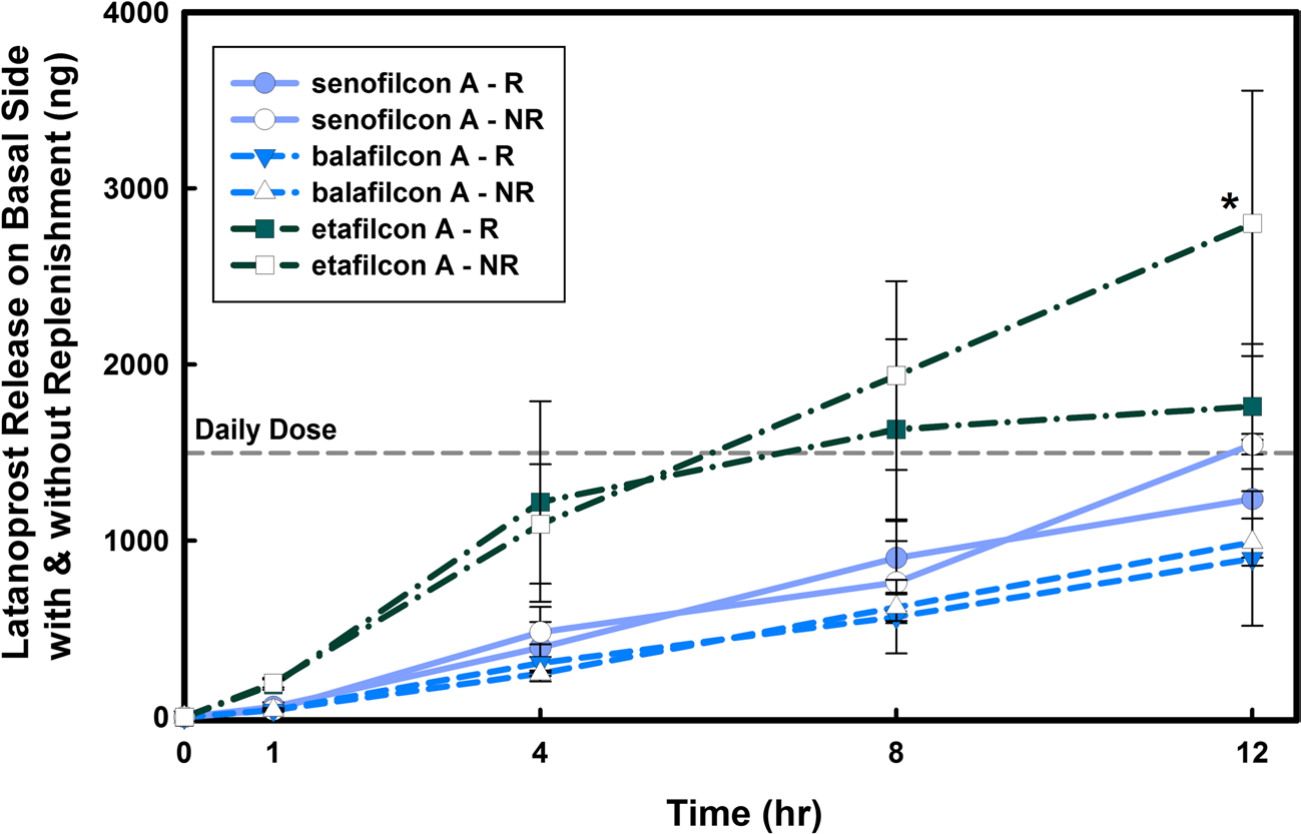
The effect of replenishment on the basal levels of latanoprost during latanoprost release from three contact lens materials in a monolayer curved cornea model. Lenses were soaked for 24 h in drug solution (123* µg/mL*) and then placed on the curved monolayer for 12 h. Aliquots were taken at specific times from the basal side and concentrations were measured using EIA. Daily dose line represents the amount of the administered latanoprost for a glaucoma patient [36]. No significant difference was observed when comparing the latanoprost release from the contact lens material and diffusion through the model to the basal side between replenishment (R) and no-replenishment (NR) conditions (*p* > 0*.*2). ^*^ Release from etafilcon A under no- replenishment condition was significantly higher at 12 h (*p* < 0*.*001). (n = 3, Mean ± SD)

The basal release results represent the amount of latanoprost that has been delivered/transported to the other side of the monolayer curved cornea models. The release results, as illustrated in Fig. 6, showed a linear increase for all contact lens materials on the basal side in both no-replenishment and replenishment conditions. The drug concentration on the basal side was similar under both conditions (*p* > 0*.*2), and the amount of latanaprost on the basal side was comparable to the prescribed clinical daily dosage.

When normalizing the total drug release (combined apical and basal drug concentrations) at 12 h to the total drug uptake into each lens material, the total drug release from the two silicone hydrogel materials (balafilcon A and senofilcon A) accounted for less than 2% in the replenishment model and less than 3% in the immersion model, while for the conventional hydrogel material (etafilcon A) total drug release was slightly above 3% and 8% of the total drug uptake respectively.

### Apical and Basal Release of Low Dose Latanoprost-Eluting Lenses in a Monolayer and Multilayer Curved Cornea Model

**Fig. 7:**
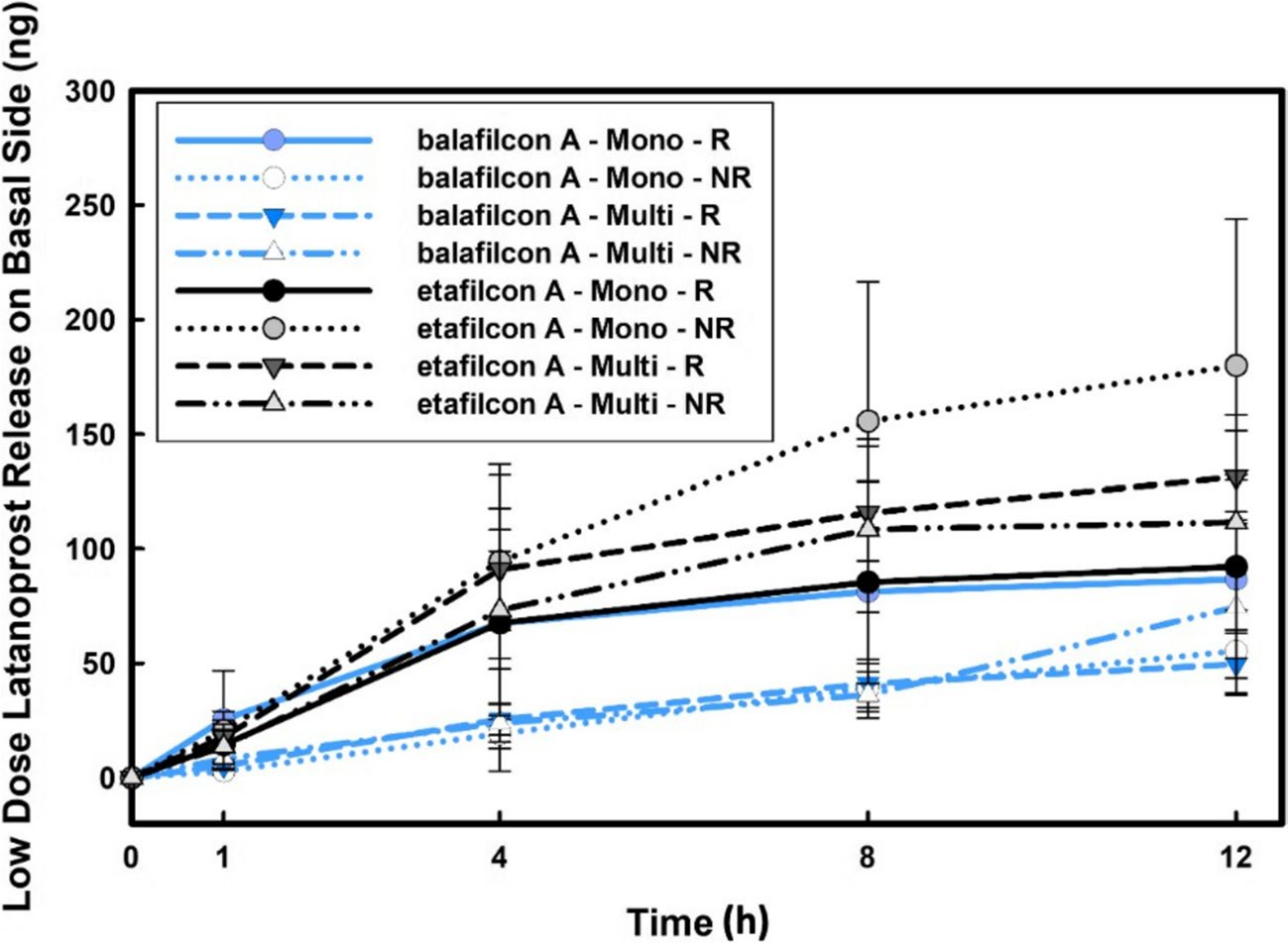
Effect of replenishment on the basal levels of latanoprost during latanoprost release (low dose) from etafilcon A and balafilcon A contact lens materials in a monolayer (mono) and multilayer (multi) curved cornea model. Lenses were soaked for 24 h in drug solution (13*.*44* µg/mL*) and then placed on the curved corneal models for 12 h. Aliquots were taken at specific times from the basal side and concentrations were measured using EIA. No significant difference was observed when comparing the latanoprost release from the contact lens material and diffusion through the model to the basal side between replenishment (R) and no-replenishment (NR) conditions (*p* = 0*.*79). (n = 3, Mean ± SD)

The high bioavailability of release and diffused latanoprost in the case of the high dose experiments (where lens uptake was around 120* µg*/lens as reported in Table 2) merited investigation of low dose latanoprost release from contact lens materials in our in vitro model. Balafilcon A and etafilcon A lenses were selected for the low dose experiments (lens uptake being around 13* µg*/lens) where the effects of replenishment as well as monolayer versus multilayer were assessed. The basal release of latanoprost, as illustrated in Fig. 7, showed a significant drop in released drug when compared to the high dose study (*p* < 0*.*001), suggesting the doping concentration as a potential factor in designing latanoprost-eluting contact lenses. While sustained latanoprost release was achieved over time, no significant differences were observed between replenishment conditions (*p* = 0*.*79). Additionally, no significant difference (*p* = 0*.*24) were observed between the monolayer and multilayer curved cornea models under either replenishment and no-replenishment conditions, further confirming our previous observations under no replenishment conditions in an onlay contact lens model [35]. Similar to the high dose release studies, the latanoprost basal release from etafilcon A was significantly higher than that of balafilcon A (*p* < 0*.*001).

### Effect of Active Transport on Basal Release of Latanaprost from Drug-Eluting Contact Lenses In Vitro

**Fig. 8:**
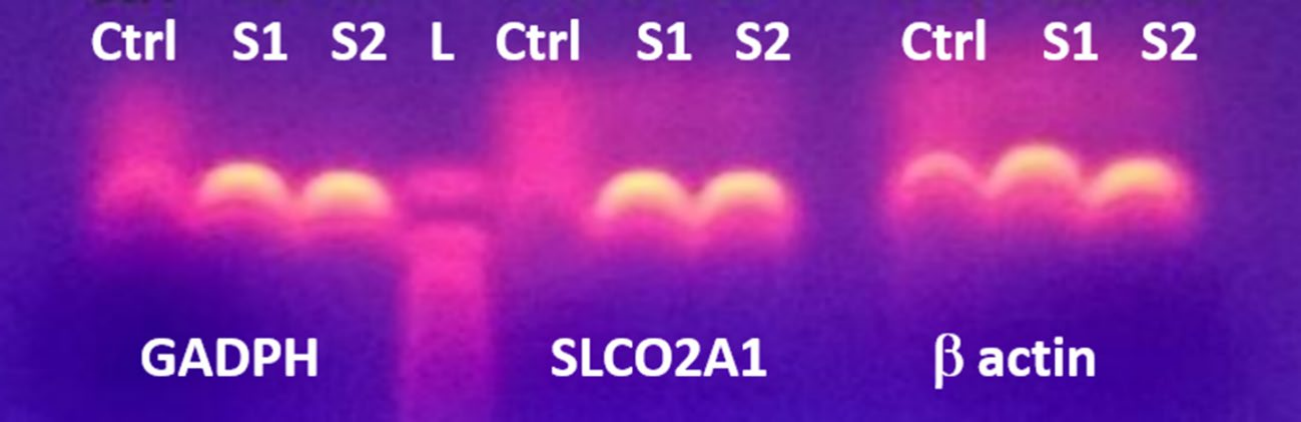
Gene expression of the prostaglandin transporters (OATP2A1/SLCO2A1) in HPV-immortalized human corneal epithelial cells. RT-PCR was performed on extracted RNA from HCEC, followed by gel electrophoresis of the amplified cDNA samples. Sample 2 is a 10X dilution of sample 1. RNA samples were used as negative control (Ctrl)

Comparison of the OATP2A1/SLCO2A1 gene to the housekeeping genes indicated that OATP2A1/SLCO2A1 cDNA was amplified (data not shown), confirming the presence of this gene in the immortalized human corneal epithelial cell line. The gel electrophoresis results from the amplified cDNA samples (Fig. 8) further demonstrated the presence of the prostaglandin transporter OATP2A1/SLCO2A1 gene in cells.

The basal release of latanoprost from balafilcon A and senofilcon A contact lens materials through live, prostaglandin transporter-inhibited (i.e. diclofenac-treated) and fixed cells was measured under no replenishment conditions in vitro. Inhibiting prostaglandin transporters in the monolayer led to a significant reduction (*p* < 0*.*001) in the basal release of latanoprost when compared to untreated cells, with a 50 ± 10% and 52 ± 10% reduction for balafilcon A and etafilcon A, respectively. Inhibiting active transport and cell metabolism through fixing cells led to a significant decrease (*p* < 0*.*0001) in the observed basal release compared to untreated cells, 83 ± 1% and 91 ± 3% reduction for balafilcon A and etafilcon A, respectively.

## Discussion

This study aimed to assess the impact of modeling tear replenishment in an in vitro cell culture model for drug delivery studies of ophthalmic materials. The curved cornea models used in these experiments have proven to be an effective platform for understanding biocompatibility of contact lens material and lens cleaning solutions [10, 34], and can see further use with a dynamic replenishment model for drug delivery investigation. While several in vitro models have recently been developed to assess drug release from contact lenses under dynamic conditions [38–40], to our knowledge, these are the first results combining tear replenishment and drug-eluting contact lenses in a corneal epithelial cell in vitro model. In the human eye, the drug released from an ophthalmic material is first dissolved in the tear film with a limited volume before it diffuses through the corneal epithelium. Our in vitro model enables to characterize the release profile and amount of drug diffusion under more physiological conditions. Furthermore, comparing the apical release between replenishment and immersion (no- replenishment) conditions as well as basal release highlighted how this dynamic in vitro cell culture model may help in gaining a better understanding of the role that tear replenishment plays in the human eye and inform the design and testing of drug delivery biomaterials.

In the case of drug release studies in a dynamic system, in comparison to the immersion/no-replenishment in vitro model, two different mechanisms may occur:

1. the in vitro drug release may be elevated compared to the immersion model; this would be due to the high solubility of the drug in the tear solution, being washed away and drained by the TRS, as it would be in tears through the lacrimal system, or
2. the in vitro drug release may decrease due to the low solubility of the drug in the tear solution resulting in lower concentration of the drug being released into a much smaller residual tear liquid, especially between the contact lens and the corneal cells, as might be the case in the tear film between the epithelium and the contact lens (post-lens tear film).

The first proposed mechanism may be the more dominant effect for hydrophilic ophthalmic drugs while the second mechanism would be the predominant effect for hydrophobic compounds such as latanoprost. The considerably high drug uptake and low drug release from the silicone hydrogel lenses can be attributed to the higher affinity of the hydrophobic latanoprost to the relatively hydrophobic silicone hydrogel material (as would be the case with proposed mechanism #2). Comparing the results obtained using the two conditions (replenishment versus no-replenishment) showed the dominant effect of the hydrophobic-hydrophobic interactions between the drug and the contact lens material, resulting in a reduced apical drug loss into the supernatant. Etafilcon A showed marginally less affinity toward latanoprost when compared to the silicone hydrogels (see Table 2). This resulted in increased drug release on the apical area and diffusion into the basal side. This is consistent with previous results [35], where a significantly higher amount of drug release was observed from high water content hydrogel contact lenses such as etafilcon A. This can be attributed to the bulk properties of such materials and their relatively lower affinity towards highly hydrophobic compounds such as latanoprost.

The significantly elevated latanoprost release on the apical side in the immersion model was in spite of the considerably lower volume of supernatant fluid in the immersion model (2*.*5* mL* vs. 12* mL*). This phenomenon can be explained if one were to consider the role that live cells play in transporting and hydrolyzing the prodrug. Previous studies proved the crucial role of live cells in latanoprost’s release from contact lens material and its diffusion through a cornea model in an immersion model [35]. Due to the hydrophobicity of the latanoprost, it may easily penetrate through the transcellular pathways into corneal cells where it will be hydrolyzed into the free-acid form. In the case of the monolayer curved cornea model, the free-acid drug may leave the cell membrane to either the apical or basal side. The limited post contact lens tear film volume present in the replenishment conditions would inhibit apical diffusion of the latanoprost free-acid, while in the immersion conditions, the apical release would be promoted. One may further hypothesize that in the multilayer curved cornea model, where hydrolysis of the prodrug may occur in lower layers, the basal or apical release of latanoprost free-acid may equally occur. Diffusion to the basal side may slightly decrease due to both the cell layer and the cell culture membrane. This may explain the higher apical release especially in the monolayer model under the no-replenish condition. While small differences might be justified by this mechanism, the significantly higher apical release in high dose latanoprost-eluting etafilcon A requires further investigation. Latanoprost is a highly hydrophobic drug, that could easily release from etafilcon A and dissolve in the apical solution in the ester form as previously has been observed in high water content hydrogels [35]. This might explain the elevated apical release of high dose latanoprost-eluting etafilcon A in the no-replenish model. It should be noted that the EIA kit used in these studies does not distinguish between the two drug forms and thus it will be difficult to verify such a hypothesis. To gain a further understanding of the drug release profile and hydrolysis of the drug in the in vitro cornea model, future studies will involve longer time release study and analytical characterization of latanoprost (ester versus acid form).

While the immortalized human corneal epithelial cells expressed the OATP2A1 transporter gene, our inhibition results with Diclofenac suggested that these transporters were only partially responsible for the transport to the basal side of the latanoprost released from the contact lens materials. The role of other active transport mechanisms and metabolizing enzymes in latanoprost permeation in our in vitro model was further highlighted by fixing the cells prior to the release experiments. Our results on the contribution of different mechanisms of latanoprost transport across cells are in accordance with other in vitro drug permeability studies using primary corneal epithelial cells [23, 41, 42] and further suggest the validity of our curved cornea models with immortalized corneal epithelial cells.

Our experimental model allowed the comparisons of apical and basal side latanoprost release profiles between different conditions, monolayer versus multilayer and replenishment versus no-replenishment. It is interesting to note that except for the etalfilcon A-loaded contact lens material, no significant differences in the levels of latanoprost delivered to the basal side was noted between experimental conditions. The absence of significant differences in delivery to the basal side between a monolayer and multilayer corneal model corroborates our prior results using non-curved corneal models [35]. Although the tear replenishment system highlighted differences in latanoprost found in the apical side, no significant changes were observed between replenishment and no-replenishment for the amount of latanoprost that was delivered to the basal side of our corneal models. Combined together, our results would thus suggest that in vitro models to assess drug delivery from biomaterials may be simplified to using a monolayer of corneal epithelial cells while still gaining relevant insights on drug delivery and transport. The impact of tear replenishment in in vitro testing may be more relevant in biocompatibility and cytotoxicity studies where accumulation of a compound may change corneal epithelial cell response. It is important to note that while tear replenishment and a curved multilayer cornea model provide a more physiological testing platform, such a model is also more resource intensive. Future work will aim to validate the model and determine how our in vitro results correlate with in vivo experiments.

While the experimental model took into consideration the replenishment environment of the ocular surface and cells, a limitation of the current in vitro model, which may also explain the limited differences between replenishment versus no- replenishment, is that it does not reproduce the mechanical stimulation that is created from the movement of the eyelid through blinking. In an in vitro model of the ocular surface, this mechanical stimulation would be important as it would allow some movement of the lens and thus promotes tear exchange in the post-lens tear film, which can also affect drug delivery kinetics [43, 44].

## Conclusion

Through modeling the microfluidics of tear replenishment in vitro, the tear replenishment system offers an ocular drug delivery testing platform with a reliable and continuous replenishment of the surface of the cell culture model that can contribute to a better understanding of the interactions between corneal epithelial cells and drug delivery systems in vitro. The latanoprost release studies further confirmed the significant role that cells play in the release of an ocular prodrug from a contact lens material. The results presented in this study also demonstrated yet another important role that a dynamic model will have in predicting the amount of drug that can be lost from a contact lens into the tear film/lacrimal system.

While our in vitro model focused on tear replenishment and provided valuable insights on drug release from commercially available lenses, our model does not yet fully represent the dynamic physiological environment of the contact lens-cornea interface as a blinking mechanism, which may affect fluid exchange underneath the contact lens, was not included. Interestingly, our results also highlighted that the in vitro curved monolayer model with no tear replenishment, a simpler cell model, could also provide relevant information on drug released from a biomaterial and transport.

Future work will aim to correlate our in vitro release results to in vivo data. Additionally further investigation is warranted as our in vitro curved cornea model with tear replenishment, which offers a more physiologically relevant ocular surface model where accumulation of released/leached compound is reduced, may provide added value to in vitro biocompatibility studies of novel drugs and ophthalmic materials, allowing to better characterize their impact on cell phenotype in vitro.

## Data Availability

The datasets generated during and/or analysed during the current study are available from the corresponding author on reasonable request.
